# Novel Alleviation Mechanisms of Aluminum Phytotoxicity via Released Biosilicon from Rice Straw-Derived Biochars

**DOI:** 10.1038/srep29346

**Published:** 2016-07-07

**Authors:** Linbo Qian, Baoliang Chen, Mengfang Chen

**Affiliations:** 1 Department of Environmental Science, Zhejiang University, Hangzhou 310058, China; 2 Key Laboratory of Soil Environment and Pollution Remediation, Institute of Soil Science, Chinese Academy of Sciences, Nanjing 210008, Jiangsu Province, China; 3 Zhejiang Provincial Key Laboratory of Organic Pollution Process and Control, Hangzhou 310058, China

## Abstract

Replacing biosilicon and biocarbon in soil via biochar amendment is a novel approach for soil amelioration and pollution remediation. The unique roles of silicon (Si)-rich biochar in aluminum (Al) phytotoxicity alleviation have not been discovered. In this study, the alleviation of Al phytotoxicity to wheat plants (root tips cell death) by biochars fabricated from rice straw pyrolyzed at 400 and 700 °C (RS400 and RS700) and the feedstock (RS100) were studied using a slurry system containing typical acidic soils for a 15-day exposure experiment. The distributions of Al and Si in the slurry solution, soil and plant root tissue were monitored by staining methods, chemical extractions and SEM-EDS observations. We found that the biological sourced silicon in biochars served dual roles in Al phytotoxicity alleviation in acidic soil slurry. On one hand, the Si particles reduced the amount of soil exchangeable Al and prevented the migration of Al to the plant. More importantly, the Si released from biochars synchronously absorbed by the plants and coordinated with Al to form Al-Si compounds in the epidermis of wheat roots, which is a new mechanism for Al phytotoxicity alleviation in acidic soil slurry by biochar amendment. In addition, the steady release of Si from the rice straw-derived biochars was a sustainable Si source for aluminosilicate reconstruction in acidic soil.

Soil amendment by adding biochar is a novel and practical approach which may replace biosilicon and biocarbon for soil amelioration and pollution remediation^1^,^2^,^3^,^4^,^5^,^6^,^7^,^8^. Biochar produced during the pyrolysis of biomass has received increasing attention as a novel material for contaminated soil remediation^2^,^4^,^9^, carbon sequestration^1^,^3^,^5^,^6^,^10^, N_2_O emission reduction^11^,^12^, and an electron shuttle for chemical or biochemical reactions^13^,^14^. Generally, biochar is long-standing in soil and thus considered as a feasible material for long-term carbon fixation^1^,^5^,^6^,^10^,^15^. By contrast to carbon fixation, the highly soluble ash and inorganic ions in biochar can easily leach out^7^,^8^,^16^. An increasing number of studies have shown that the inorganic components in biochar, e.g., Fe, P, Ca and Si, played crucial roles in the immobilization of heavy metals^17^,^18^,^19^,^20^. Therefore, the fate of the inorganic ions in biochar may affect the soil pollutant removal and the long-term function of biochar in the environment^15^,^16^,^20^.

Soil acidification has received increasing attention because of its seriously adverse effects to plants and environment. Unfortunately, the acidic soil occupies approximately 40% of the world’s arable land and is extending everyday^21^,^22^,^23^,^24^. Typically, desilicification is the primary reason of soil acidification. In addition, many arable lands in the world suffer from silicon depletion and aluminum toxicity, particularly acidic soil^25^,^26^,^27^. The application of biochar amendment to the amelioration of acidic soil has prompted concerns^4^,^28^,^29^. Based on 103 independent statistical analysis studies, Liu *et al*. have demonstrated that the crop productivity was increased by 30% with biochar application to acidic soils (pH < 5)^28^. Aluminum (Al) toxicity is a main factor limiting plant productivity in acidic soils; therefore, the Al phytotoxicity alleviation is considered as a key process for acidic soil amelioration^27^,^30^. Recently, Chen and co-workers initially demonstrated that biochar amendments effectively reduced Al phytotoxicity due to the biochar’s liming effect and adsorption properties^4^ and that the surface adsorption and coprecipitation of Al with silicate particles to fix Al in soil^4^,^29^. However, the unique roles of silicon (Si)-rich biochar in the long-term alleviation of Al phytotoxicity have not been discovered. The existence of “*Terra preta*” in the Amazon and similar char-amendment soils elsewhere in the world provide important examples of the long-term acid soil amelioration by biochar^31^,^32^. The loss of Si during soil acidification by aluminosilicate decomposition is the primary cause of Al toxicity. Rice straw contains approximately 10–20% Si, which is an ideal feedstock for the preparation of Si-rich biochars^15^,^33^,^34^. A recent study has indicated that the Si release from rice straw-derived biochars could be a sustainable Si source^33^. The transformation, morphology and dissolution of Si in rice straw-derived biochars were highly dependent on the pyrolytic temperature^33^. The dissolution of Si in the biochars increased with the increasing pyrolytic temperature (up to 700 °C). Below 700 °C, Si in biochars is mainly in the amorphous phase^15^,^29^,^33^. Therefore, the effects of released biosilicon from different biochars to Al toxicity alleviation in acidic soil require further elucidation.

In this study, the alleviation of Al phytotoxicity to wheat plants (root tips cell death and root elongation) by rice straw-derived biochars pyrolyzed at 400 and 700 °C (RS400 and RS700) was investigated using a slurry system containing typical acidic soils (oxisol) in a 15-day exposure experiment. Meanwhile, the Al and Si distributions in the soil, water, and plant root tips were monitored using hematoxylin and morin staining, a chemical extraction method, and scanning electron microscopy. A novel alleviation mechanism of Al phytotoxicity by released biosilicon from biochar is proposed for the sustainable application of biochars.

## Results and Discussion

### Effects of biochar amendment on plant growth and root elongation

**Figure 1:**
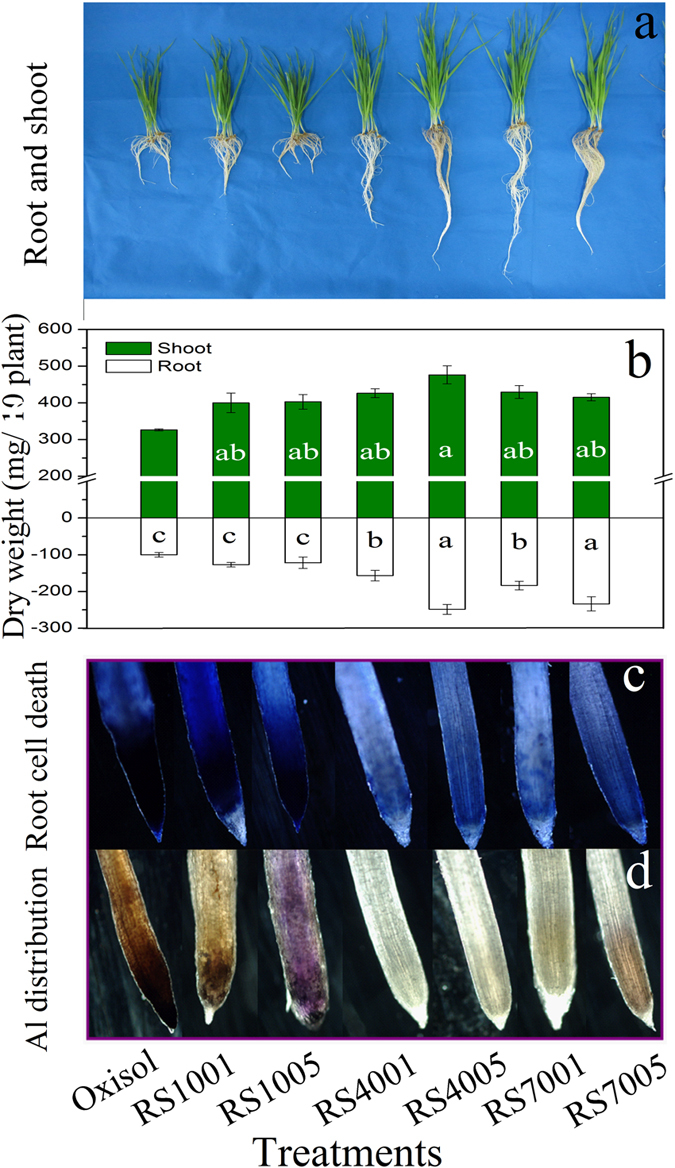
Effects of acidic soil slurry (oxisol), rice straw biomass (RS100), and biochars (RS400 and RS700) on wheat seedling growth (**a**), root and shoot dry weight (**b**), root tip cell death (**c**) and Al distribution on the root tip (**d**). The first three numbers in the sample names represent the pyrolysis temperatures, and the last number represents the amendment sample percentage.

The plant growth as well as the weight of the root and shoot tissues of wheat seedlings exposed to oxisol slurry with and without the presence of biomass (RS100) and biochars (RS400 and RS700) are presented in Fig. 1a,b. After the 15 d exposure experiment, the root elongation of wheat was significantly inhibited by the oxisol amendment, and the weight of the wheat root was only 100 mg/10 plants for the oxisol slurry without biochar or biomass amendment (Fig. 1b). Crop growth restriction is a common phenomenon caused by oxisol^35^, which is related to the phytotoxicity of Al released from oxisol^30^. After adding 1% rice straw (RS1001), the weight of the wheat roots increased to 127 mg/10 plants, and the weight of the shoot increased from 326 mg/10 plants (oxisol) to 400 mg/10 plants (RS1001). The increased weight of root and shoot could be attributed to the nutrition provided by the added rice straw. This result is consistent with the previous report revealing that rice straw can promote the crop growth^36^,^37^. When the amount of rice straw was increased to 5%, the root and shoot length were not further increased as compared with RS1001. When the rice straw-derived biochars were added to the oxisol slurry, the wheat growth was significantly improved. After the addition of 1% RS400, a significant elongation of the wheat root was observed (Fig. 1a), the root dry weight reached 156 mg/10 plants, and the shoot dry weight increased to 426 mg/10 plants, indicating a significant growth promotion by RS4001 addition. When the addition amount of RS400 was increased to 5%, the elongation of the wheat roots was further promoted (Fig. 1a), and the weight of the roots and shoot increased to 249 mg/10 plants and 476 mg/10 plants, respectively. These observations suggested that RS400 was effective for the alleviation of the root growth inhibition by oxisol, and the plant growth was promoted with the increase of the biochar addition. The amendment of RS700 at 1% and 5% showed a similar trend to that of RS400, which confirmed that biochars can be used as effective materials to ameliorate the plant root growth inhibition in acidic soil, such as oxisol.

### Alleviation effects of biochar amendment on wheat root tip cell death

The integrity of the root tip plasma membrane after 15 d of exposure to oxisol slurry were evaluated by the Evans blue staining approach. The death of the root cell in the wheat root tips with and without the presence of biomass and biochar is shown in Fig. 1c. The root cell death was clearly observed in oxisol slurry, and the biomass (rice straw) amendment could not alleviate the wheat root cell death. Al toxicity is a primary factor limiting plant growth in acidic soils, and the root tips are the main target sites of the dissolved Al^4^,^30^. Delisle *et al*. have reported that Evans blue will display a strong adsorption when the root tips were destroyed by Al toxicity^38^. Interestingly, after biochar amendment, the root tips cell death were significantly reduced, which indicated that biochar can alleviate Al phytotoxicity to the root tips in an acidic slurry. The distribution of Al in the wheat root tips observed by the hematoxylin staining (Fig. 1d) confirmed that the root tips cell death were caused by the Al toxicity. As shown in Fig. 1d, when the root tips were treated by oxisol, a light purple coloration was observed, indicating that an Al complex was formed; and a deeper purple coloration was observed after RS1005 treatment, confirming that more Al complex was formed. In order to confirm that the root cell death and Al distribution of wheat seedlings was representative, another group of their staining results were provided in Figure S1. This phenomenon was correlated to the measured Al concentrations in the root tips (Fig. 1c). Without biochar amendment, the wheat root tips contained a substantial amount of Al, and the root tips cell growth were deformed. A previous study has reported that hematoxylin could form a complex with Al, and the hematoxylin content increased as the Al concentration increased^39^. After the biochar amendment, the Al distribution in the wheat root tips was significantly decreased. The amendment of biomass and biochar to oxisol exhibited different effects on the alleviation of Al phytotoxicity^40^,^41^. The amendment of biomass showed only a slight effect on Al phytotoxicity alleviation, while the addition of biochar effectively alleviated the Al phytotoxicity of oxisol.

**Figure 2:**
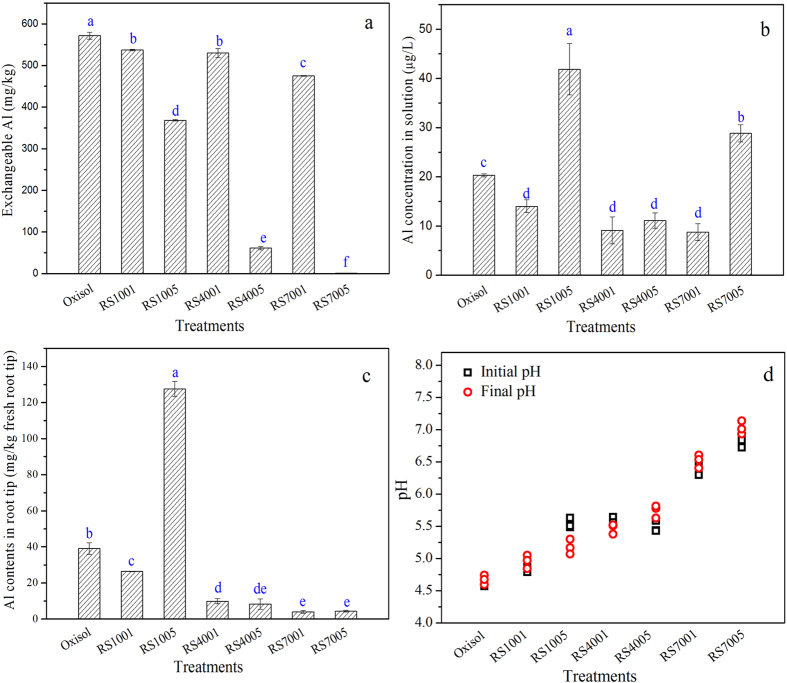
Effects of acidic soil slurry (oxisol), rice straw biomass (RS100), and biochars (RS400 and RS700) on the Al contents in exchangeable Al in oxisol (**a**), culture solution (**b**), the root tips (**c**) and pH change (**d**).

To further understand the Al phytotoxicity alleviation by biochar, the Al migration in the system containing the oxisol slurry and the plant was studied. The soil exchangeable Al, Al concentration in solution and Al content in the wheat root tips were measured (Fig. 2). The soil exchangeable Al content represents the available Al that may be toxic to the plants. The addition of rice straw and biochars reduced the soil exchangeable Al, which was consistent with a previous study^40^. Notably, amendment of 5% biochar significantly reduced the soil exchangeable Al. After the addition of 5% RS400, the soil exchangeable Al concentration reduced to 61.3 mg/kg soil, which was only 10% that of oxisol (572 mg/kg). Although the pH of RS1005, RS4001 and RS4005 treatments were all around 5.5, the exchangeable Al concentrations were 368, 530 and 61.3 mg/kg, respectively, indicating the available Al was decreasing with the increased amount of biochar.

After applying 5% of RS700, the exchangeable Al concentration in the soil was only 1.09 mg/kg soil. Obviously, the amendment of 5% biochar can significantly reduce the soil exchangeable Al. The changes in Al concentrations in the slurry solution before and after biochar amendment displayed a trend different to the soil exchangeable Al concentration. After the addition of RS1001, RS4001, RS4005 and RS7001, the Al concentration in the solution was significantly decreased (Fig. 2b), but both RS7005 and RS1005 did not decrease the Al concentration in the solution. Although the Al concentrations were high for the treatment of RS7005, the solution pH was increased to 7.0, and the primary Al species was Al(OH)_4_^−^, which has a low toxicity to plants^4^. The liming effect of biochar could elevate the soil pH, thus alleviating the Al phytotoxicity^1^,^5^.

In Fig. 2, the solution pH value was significantly increased, especially after the amendment of RS700. The solution pH was approximately 5.5 with the addition of RS400, and the Al concentration in the solution was lower than 10 μg/L. Remarkably, the solution pH of RS1005 was also around 5.5, but the Al concentration in the solution was increased to 41.9 μg/L. Reasonably, the lower Al concentration of RS400 was primarily due to the Al adsorption by biochar. The soil exchangeable Al and solution Al concentrations were lowered after biochar amendment, and the liming effect and Al adsorption contributed to the Al phytotoxicity alleviation^29^. Previous report has proved that the biochar was effective for Al adsorption, and the maximum adsorption capacity of RS400 reached up to 398 μmol/g, which is about three times higher than that of the rice straw (131 μmol/g)^29^. The Al adsorption amount of biochar (RS400) was also higher than that of kaolinite (about 250 μmol/g)^42^.

The Al concentration decreased by biochar amendment was confirmed by the results of the Al concentration in the root tips. Without the addition of rice straw and biochar, the Al concentration in the root tips was 39.1 mg/kg fresh root. With 1% RS100 amendment, the Al content in the root tips was 26.4 mg/kg fresh root, which was lower than that of oxisol, indicating that the rice straw can alleviate Al toxicity to a certain extent. However, the Al content in the root tips was obviously increased to 128 mg/kg fresh root with the 5% RS100 amendment. The primary reason for this result was that the high amount of RS100 promoted the dissolution of Al and then increased the Al accumulation in the plant root. Previous studies have shown that dissolved organic matters in soil can elevate the availability of metals^40^. By contrast, the addition of biochar (RS400 and RS700) significantly lowered the Al concentration in the root tips. For the amendment treatments of RS4001, RS4005, RS7001 and RS7005, the Al concentrations in the root tips were 9.91, 8.30, 3.95 and 4.36 mg/kg fresh root, respectively. Obviously, the addition of biochar can significantly reduce the root uptake of Al. Plant growth is the premise for its absorption of other mineral nutrients (such as Si), then significantly reduced the Al content of root. Plant growth also helps alleviate Al toxicity inside the plants via synthesis of Al-Si compounds *in vivo*, which will be elucidated below.

### Effects of biochar amendment on the root cross-section structure and Al distribution

**Figure 3:**
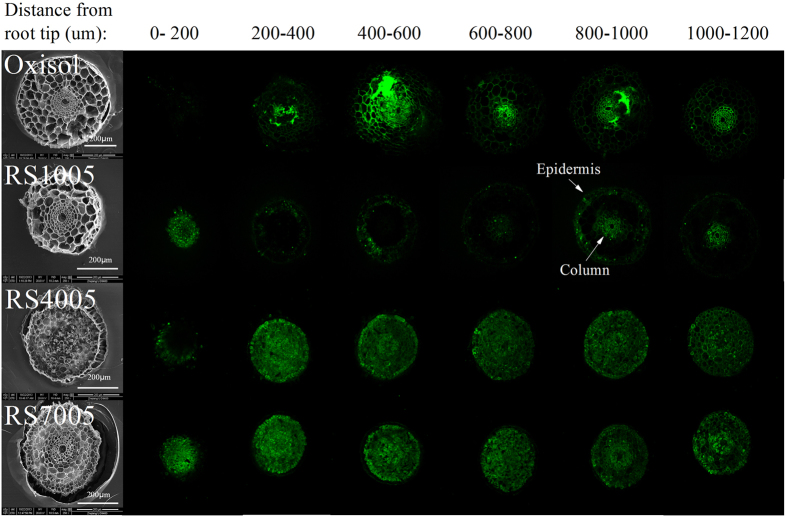
Effect of acidic soil slurry (oxisol), rice straw (RS100), and biochars (RS400, RS700) on the root structure (SEM, left-hand side) and the *in-situ* Al distribution of the cross-section of the root tips with different lengths (right-hand side). Note that the Al distribution on the cross-section of the epidermis was relatively low, while the Al accumulation in the column was relatively high.

The effect of biochar on the cross-section structure of the wheat root is presented in Fig. 3. The root cross-section structures of wheat exposed in the treatments of oxisol and RS1005 were damaged. Meanwhile, the root tips without the meristematic zone indicated that the root tips stopped growing. After amendment of biochar (RS400 and RS700), the root tips cells grew well with the meristematic zone. The *in-situ* Al distribution in the cross-section of different root lengths with morin staining is also presented in Fig. 3.

Without biochar, as the distance increases from near the root tips to far away from the root tips, the high Al accumulation in the root tips transfers from the epidermis to the column. Al accumulation in certain areas was higher than in the part around the root tips, which was the evidence of cell damage. The RS1005 showed the same trends as oxisol. After the addition of biochars (RS400 and RS700), the integrity of the root tips was recovered and grew well. However, surprisingly, the Al concentrations on the cross-section of the root tips ranging from 0–1200 μm were significantly higher than those of the treatment without biochar amendment. Then, as the distance from the root tips increased, the Al concentrations gradually decreased. Furthermore, the Al distribution on the cross-section of the epidermis was relatively low, while the Al accumulation in the column was relatively high. These observations demonstrated that with biochar amendment, Al penetrates the root tips as the plant grows, but the Al phytotoxicity was significantly alleviated. This contradictory result cannot be explained by the short-term alleviation mechanisms, such as biochar’s liming effect and adsorption properties^4^. Noting that the root tips of RS1005 by haematoxylin staining were positively identified Al (Fig. 1d), while in Fig. 3 Morin fluorescence shows the least Al. This distinct observation is attributed to the difference of haematoxylin staining and morin staining methods monitoring the Al distribution in the different locations of root tips cells^43^. The haematoxylin staining was developed to probe Al in the cell wall and vacuole of plant root tissues, while the morin staining was used to probe Al in the cytoplasm. As the root tips of RS1005 without meristematic zone, the Al mainly concentrated on cell wall, so the haematoxylin staining (Fig. 1d) showed high Al concentrations existed.

### Novel alleviation mechanisms of Al phytotoxicity in oxisol by biochar

**Figure 4:**
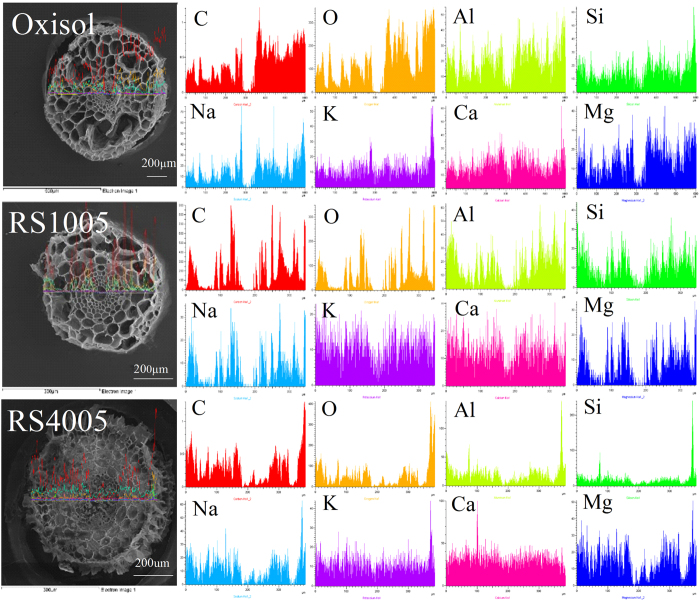
Effect of acidic soil slurry (oxisol), rice straw (RS100), and biochars (RS400) on the elemental mapping of the root tips. The elemental mapping was derived from the line scan of SEM-EDS.

**Figure 5:**
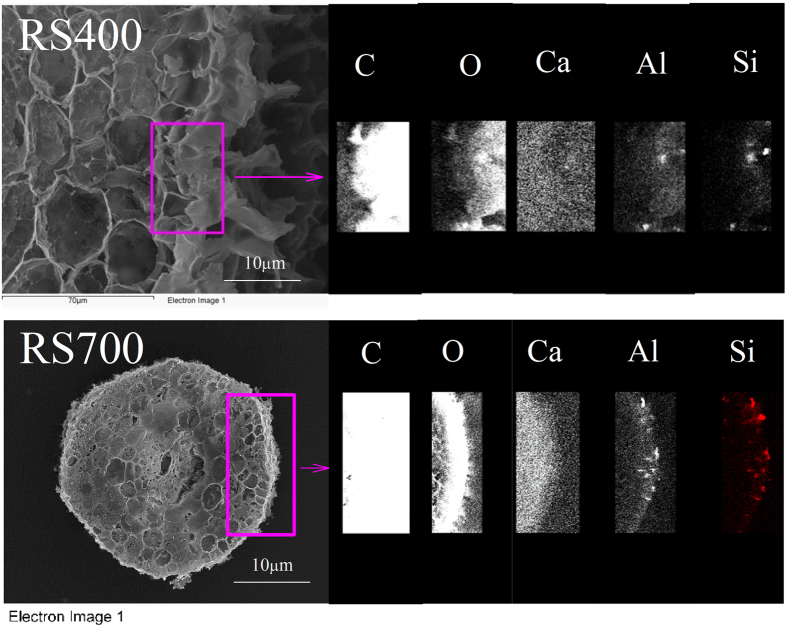
SEM-EDS of the elemental distribution of the cross-section of the root tips exposed to oxisol slurry in the presence of biochars (RS400 and RS700).

To discover alleviation mechanisms of Al phytotoxicity from inside of the plant, the elemental mappings of the root tips cross-section without and with biochar amendment were studied by SEM-EDS (see Figs 4 and 5). The root tip cross-section was rich in C and O as well as other nutrition elements such as Ca, K, Na, and Mg. Among the treatments of oxisol, RS100 and RS400, the composition of C and O showed a strong consistency, which is primarily because the root is composed of hydrocarbons. Interestingly, after RS400 amendment, the nutrition elements including Ca, K, Na, and Mg in the root tips increased, especially Ca. Previous studies have shown that Al can inhibit Ca uptake in the plant roots^44^. Al and Si were also determined. Among the treatments of oxisol, RS100 and RS400, the Al and Si distribution maps exhibited a strong consistency with the amendment of RS400, i.e., colocalization of Al and Si. In order to confirm that this phenomenon was representative, two additional groups of the elemental mapping of the root tips (oxisol and RS400) were provide in Figure S2. Furthermore, the concentrations of Al and Si were high in the root epidermis because of the possible formation of the alumina-silica compound by Al and Si in the root tip epidermis. Because the rice straw biochar contained a substantial amount of Si components, obvious Si dissolution was probable when rice straw-derived biochar was added to the soil. Recent studies have reported that a high amount of Si was released from rice straw-derived biochar^33^. In addition, the formation of the alumina-silica compound in the solution and in the plant root tips has been reported^45^,^46^,^47^,^48^. Exley and coworkers first demonstrated that the formation of hydroxyaluminosilicate (HAS) limited the biological availability of Al^49^, and the inorganic chemistry of HAS and their role in the biogeochemical cycle of Al was excellently elucidated^47^,^48^,^50^. Similarly, the intracellular Si-Al biointeraction and nanometer-scale colocalized of Si and Al via elemental mapping were used to illustrate the avoidance of Al toxicity in freshwater snails^51^.

Based on the spectral scan shown in Fig. 5, the root tip epidermis was rich in C, O, Ca, Si, and Al in which the C, O, and Ca in this area are uniformly distributed, and the distribution of Al and Si was consistent. Therefore, this result confirmed the formation of the Al-Si compound in the root tips. The Al-Si compound is an important part of the soil. Previous reports have shown that volcano ash contained substantial amounts of Si, and the ash deposition process easily formed the Al silicate compound with Al, thus reducing the Al toxicity to plants^45^. Jugdaohsingh *et al*. reported that small colloidal silica particles avidly binds Al, in the form of aluminosilicates, thus reducing its availability^46^. Hodson *et al*. have observed through X-ray microanalysis that Al and Si coexist in the Norway spruce root epidermal cells, and this may be the mechanism by which Si alleviates Al toxicity to sorghum roots^52^,^53^.

After the 15 d cultivation experiment in this study, the presence of oxisol significantly inhibited the growth of the wheat roots. A substantial number of wheat root tip cells died in the oxisol treatment, and the root cell stopped differentiating, which are common symptoms consistent with Al toxicity to plants^52^,^53^. Using Evans blue and hematoxylin staining, the area of root cell death and Al distribution were highly consistent, which confirmed that Al toxicity was the factor for the inhibition of root growth. Note that the addition of rice straw (RS100) and biochars (RS400 and RS700) showed substantial differences in Al toxicity alleviation in wheat roots. With RS100 amendment, Al toxicity in wheat roots was not significantly alleviated, and the toxicity effect was even aggravated when the added amount of RS100 increased. This result may be attributed to the release of the dissolved organic matter from the RS100 amendment, thus enhancing the migration of Al to the roots. The enhanced mobility of the contaminants by dissolved soil organic matter has been widely reported^39^. Therefore, the traditional method of returning rice straw to the field for soil nutrients can increase the risk of Al transfer to plants.

**Figure 6:**
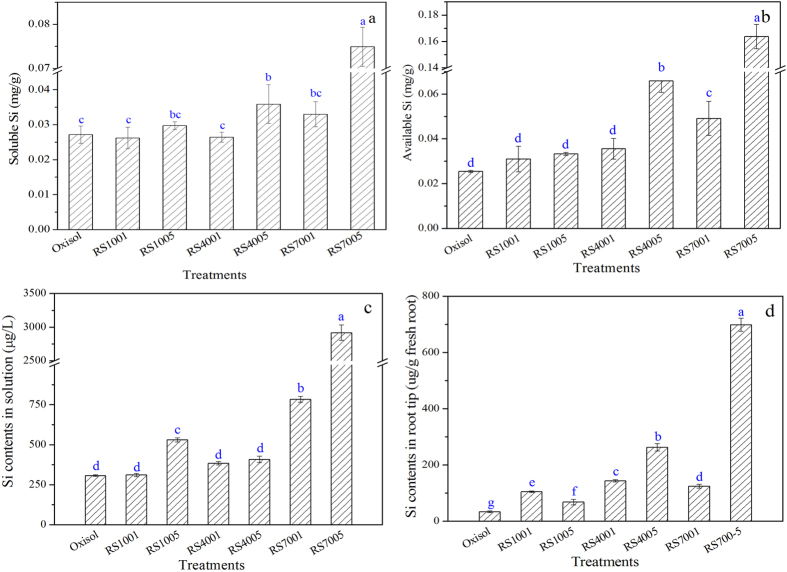
Effect of acidic soil slurry (oxisol), rice straw (RS100), and biochars (RS400 and RS700) on soil soluble Si (**a**), soil available Si (**b**), Si in the slurry solution (**c**) and wheat root tips (**d**).

After the addition of biochars, the root inhibition by oxisol was alleviated. However, the morin staining and the SEM-EDS characterization found that Al was accumulated in the root tips after the RS400 amendment. A further observation showed that the Al and Si colocalized at a specific site in the root tissue. To support the role of dissolved biosilicon from rice straw-derived biochar in the alleviation of Al phytotoxicity, the soil soluble Si contents were determined after the addition of RS100, RS400 and RS700 to the oxisol slurry (Fig. 6). The soil soluble Si content was significantly increased by RS7005, RS4005 and RS7001, which suggested that the rice straw biochars functioned as a carbon-rich and a Si-rich material. The Si contents of rice straw-derived biochars increased with the pyrolytic temperatures and the speciation of Si in biochars was dominated by amorphous Si at the pyrolytic temperature less than 700 °C^29^,^30^,^31^,^32^,^33^. Furthermore, higher temperature would also remove organic (carbon) matter and make Si more available/labile. The soluble Si content in the slurry increased in the order of RS700 > RS400 > RS100, and a similar trend was observed for the soil available Si. The content of the available Si dominated the Si availability to plants. Without the RS100 and biochars amendment, the Si content in the root tips was only 33.6 mg/g fresh root. After the addition of 1% RS100, RS400 and RS700, the Si contents in the root tips were 105, 144 and 125 mg/g fresh root, respectively. When the addition amount of biomass and biochars increased to 5%, the Si contents in the root tips reached up to 63.1, 263 and 698 mg/g fresh root with the amendment of RS100, RS400 and RS700, respectively. The biochar amendment significantly increased the soil soluble Si and available Si, thus enhancing the absorption of Si by the wheat root. The Si and Al can produce a stable complex in the plant root tips, thus alleviating Al toxicity^52^,^54^. The effects of other environmental factors, such as dissolved organic carbon, the other elements (e.g., Fe, P, and Ca) and the Si cycle in the ecosystem, on Al phytotoxicity alleviation via biochar amendment should be investigated in the future.

### Environmental implications

**Figure 7:**
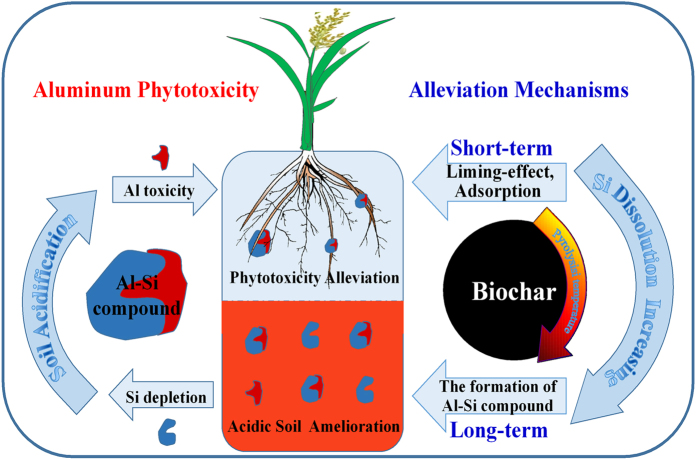
Alleviation mechanisms of Al phytotoxicity using biochar amendment with short-term effects and long-term effects.

Si and Al are the second and third most abundant elements of the Earth’s crust after oxygen. In general, they are stable in the soil in the form of aluminosilicate mineral. In acidic environments, the primary process is desilicification (Fig. 7). In fact, Al accumulation and the leaching loss of Si during soil acidification processes occur simultaneously, which leads to many arable lands in the world suffer from Si depletion and Al toxicity. Therefore, amendment of Si could be a potential way to reconstruct aluminosilicate compound with Al, thus to alleviate Al phytotoxicity and further fundamentally solve the problem of soil acidification. The current study showed that the biosilicon of rice straw biochars serves a dual role for Al phytotoxicity alleviation in acidic soil slurry by reducing the solution Al concentration because of Si particles within the biochar and promoting the formation of the Al-Si compound in the plant root tissue (Fig. 7). Reduction of the soil exchangeable Al concentration by adsorption of biochar is a short-term alleviation mechanism for Al phytotoxicity, while the novel alleviation mechanism via the formation of Al-Si compounds in the epidermis of wheat roots should be a long-term effect (Fig. 7). Furthermore, the high amount and slow release of biosilicon from rice straw biochars could be a potentially sustainable Si source for aluminosilicate reconstruction in acidic soil and plant tissue. These findings prompt further studies regarding the potential of high-Si biochar amendment for the resistance to chemical weathering and for ameliorating soil acidification and Al phytotoxicity. This process may be part of the reason for the formation of the *Terra Preta* soils in the Amazon over the 2500-year period.

## Materials and Methods

### Preparation of biochars

Rice straw (RS) was collected as precursory biomass material because returning the straw to the field is a common practice in China. The biochars were prepared by charring RS at varying temperatures; details on sample preparations are reported elsewhere^29^. Biochars produced at 400, and 700 °C are here labeled respectively as RS400, and RS700. The biomass dried at 100 °C (RS100) was used a precursory feedstock. The biomass and biochars were passed through a 0.154 mm sieve prior to use. Samples were analyzed for their elements (Na, K, Ca, Mg and Si), Fourier transform infrared spectroscopy, scanning electron microscopy spectra and X-ray Diffraction in our previous report^16^,^29^,^33^.

### Plant growth and cultivation in oxisol slurry

For high sensitivity of wheat to Al toxicity, the wheat of Yangmai 12 was used in the experiment. The germination and plant growth were treated according to a previous report^4^. After approximately 4 days of culturing in hydroponic solution without Al or oxisol soil, the plants having a root of approximately 5 cm were selected for the exposure experiment. The experiment was evaluated through a soil slurry system, which was conducted in a 500 mL polypropylene cup that contained 500 mL distilled−deionized water and 10 g oxisol. Thirty plants (10 plants in triplicate) were prepared for each treatment and the plants were randomly assigned to each treatment group.

To determine the effects of rice straw (RS100) and biochars (RS400 and RS700) on plant growth, the addition of 1% and 5% rice straw or biochars to the oxisol was performed. In most field studies, biochar amendment dosages about 0–2.7% (0–10 t/ha) and 2.7–8.1% (10–30 t/ha) were selected^28^. Thus the representative biochar concentrations (1% and 5%) in this study was reasonable to explain the benefits of biochar. Correspondingly, the exposure experiment system in which rice straw and biochar samples were amended are hereafter referred to as RS1001 and RS1005, RS4001 and RS4005, RS7001 and RS7005, respectively. The soil slurry without rice straw and biochars is referred as oxisol. A basal ratio of N:P:K of approximately 150:40:47 mg/kg of oxisol in the forms of CO(NH_2_)_2_ and KH_2_PO_4_ was initially supplied. After 15 d of cultivation, the wheat seedlings were harvested for measurement of the root and shoot weight, as well as the cell death of the root tips. Cell death was measured spectrophotometrically as the uptake of Evans blue to monitor the loss of plasma membrane integrity^4^. The experiments were replicated 10 times for each treatment to make the cell death results more representative. The supernatant of the slurry was filtered through a 0.22 μm pore-size Millipore filter for Al and Si determination. The supernatant concentrations of Al and Si were determined by a Perkin Elmer Analyst 700 atomic adsorption spectrometer equipped with a HGA-800 graphite furnace (PE700, USA). The wavelengths used for Si and Al were 251.6 nm and 309.3 nm, respectively, and the individual standard material used for Si and Al was Na_2_SiO_3_ and AlCl_3_. Finally, the residual solid was vacuum-dried and passed through a 0.154 mm sieve before its use in the exchangeable Al, soluble Si and available Si determination. The data obtained was statistically analysed by the SPSS software, version 19 (SPSS Inc., Chicago, USA). Data of root and shoot weight, Al and Si concentrations were analyzed using a one-way analysis of variance (ANOVA) with least significant difference (LSD) post hoc test. The error bars are standard deviation, and the figures were labeled with statistical significance signs (e.g., a,b,c) in the bars after the statistical tests.

### Distributions of Al and Si in a cross-sectional sample of root tips

After 15 d of cultivation, the wheat roots were washed with distilled−deionized water to remove surface impurities. Then, the root tips (approximately 1 cm) of ten plants from each treatment was cut with a knife, placed in a centrifuge tube with distilled water and washed again. Simultaneously, the agar solution was prepared by the addition of 3.00 g agar in 100 mL distilled water. After mixing and heating in the microwave until completely dissolved, the solution was cooled to 60–70 °C and poured into a petri dish. Then, the prewashed root tips was carefully transferred with tweezers to the agar dish and completely submerged by agar. After the agar solidified, the agar with the root tips was then sliced with a vibrating slicer (Leica-VT1000s). The slice was 200 μm thick. Then, the sample was freeze-dried for scanning electron microscopy-energy dispersive spectrometry (SEM-EDS, FEI Quanta 3D FEG) characterization to monitor the distribution of Al and Si. Meanwhile, the Al distributions of the cross-section in the wheat root tips by morin staining were determined by a modified method^55^. The root tip cross-sectional slices were placed in 5 mmol/L ammonium acetate buffer solution (pH 5.0) and washed for 10 min, then the root tissue was transferred to the solution containing 5 mmol/L ammonium acetate buffer and 100 μmol/L morin (pH 5.0) for staining for 1 h. After rinsing with 5 mmol/L ammonium acetate buffer solution (pH 5.0) for 10 min, the Al distribution on the root tip cross-sectional slices was observed using a laser scanning confocal microscope (LSM 710, Zeiss) with an excitation wavelength of 420 nm and an emission wavelength of 510 nm. The Al and Si distributions by SEM-EDS and the Al distribution by morin staining of cross-section of the root tips were replicated 10 times for each treatment.

### Al determination

Al concentrations in the root tips, soil exchangeable Al and Al distribution of wheat root by qualitative staining were determined. (i) Al in the root tips was determined using a modified method^56^. After 15 d of cultivation, the wheat root tips (1 cm) were cut with a knife, then washed with 0.5 mmol/L CaCl_2_ (pH 4.5) three times. The root tip was transferred into 1.5 mL centrifuge tubes containing 1 mL of 2 mol/L HCl and extracted for 24 h. Then, the supernatant was filtered through a 0.22 μm pore-size Millipore filter for Al determination. (ii) Soil exchangeable Al was determined by the KCl extraction method^33^. The concentration of KCl was 1 mol/L, and the soil-to-liquid ratio was 1:10. After mixing the soil and the KCl solution, the mixture was agitated on a reciprocating shaker at 120 rpm for 30 minutes. After the 30-minute centrifugation, the supernatant was filtered through a 0.22 μm pore-size Millipore filter for Al determination. The sample was extracted twice. Then the supernatant Al after extracted from root tips and soil were determined by a Perkin Elmer Analyst 700 atomic adsorption spectrometer equipped with a HGA-800 graphite furnace (PE700, USA). The wavelengths used for Al was 309.3 nm, the individual standard material used for Al was AlCl_3_, the analytical blanks was 0.2% HCl, and each analytical was triplicated. (iii) Al distribution in the wheat root was determined by qualitative staining^57^. After cultivation for 15 d, the root tissue was washed for 30 minutes in distilled water to remove the residual nutrient, and then the 1-cm root tip was cut and stained in 0.2% hematoxylin and 0.02% KI solution for 30 minutes. Then, the root tissues were washed with distilled water for 30 minutes to remove the residual hematoxylin. The stained root tips were directly photographed using a microscope (Eclipse E600, Melville, NY, USA).

### Si determination

The Si in the root tips (1 cm) was determined after cultivation for 15 d. The root tip was washed three times with 0.5 mmol/L CaCl_2_ (pH 4.5) and extracted for 24 h in the 1 mol/L HCl and 2.3 mol/L HF (1:2, V/V) solution. The supernatant was filtered through a 0.22 μm pore-size Millipore filter for Si determination. The total Si concentration was determined using atomic adsorption spectrometer. Meanwhile, the soil soluble Si and soil available Si were measured according to the reported method^33^. Briefly, the soluble Si was extracted using 0.02 mol/L CaCl_2_ and the available Si was extracted using the 1 mol/L HAc-NaAc (pH = 4.0) buffer method. After extraction, the supernatant concentration of Si was determined by the Perkin Elmer Analyst 700 atomic adsorption spectrometer equipped with a HGA-800 graphite furnace. The wavelengths used for Si was 251.6 nm, the individual standard material used for Si was Na_2_SiO_3_, the analytical blanks was 0.2% HCl, and each analytical was triplicated.

## Additional Information

**How to cite this article**: Qian, L. *et al*. Novel Alleviation Mechanisms of Aluminum Phytotoxicity via Released Biosilicon from Rice Straw-Derived Biochars. *Sci. Rep.* **6**, 29346; doi: 10.1038/srep29346 (2016).

## Supplementary Material

Supplementary Information
