# Supplementary material for: Novel Alleviation Mechanisms of Aluminum Phytotoxicity via Released Biosilicon from Rice Straw-Derived Biochars
Source: Sci Rep. 2016 Jul 7;6:29346. doi: 10.1038/srep29346 (PMC4935849; doi:10.1038/srep29346)
Supplement: Supplementary Information [file srep29346-s1.pdf]

Supporting Information Section for *Scientific Reports*

Novel Alleviation Mechanisms of Aluminum Phytotoxicity via Released Biosilicon from Rice Straw-Derived Biochars

Linbo Qian[1,2], Baoliang Chen[1,3]* and Mengfang Chen[2]

1. Department of Environmental Science, Zhejiang University, Hangzhou 310058, China.

2. Key Laboratory of Soil Environment and Pollution Remediation, Institute of Soil Science, Chinese Academy of Sciences, Nanjing 210008, Jiangsu Province, China.

3. Zhejiang Provincial Key Laboratory of Organic Pollution Process and Control, Hangzhou 310058, China

Supporting Information consists of 3 pages, including this one.

Feb. 16, 2016

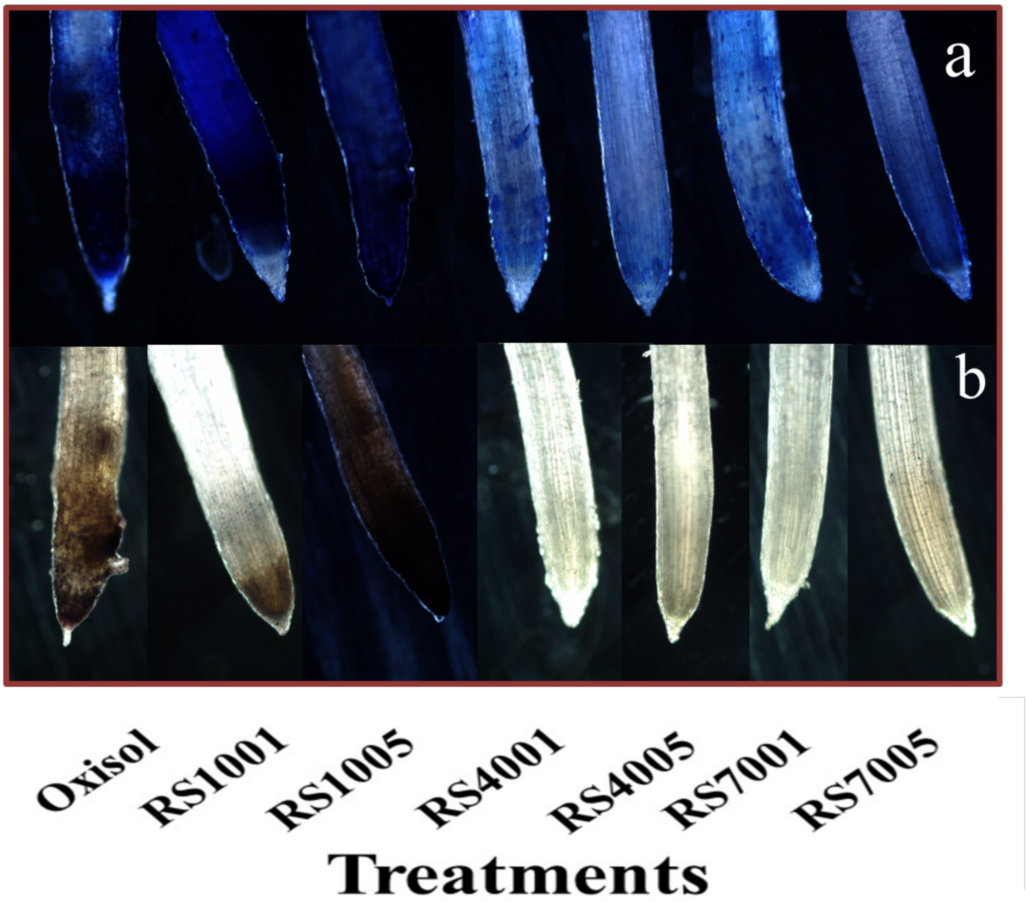

Figure S-1. Effects of acidic soil slurry (oxisol), rice straw biomass (RS100), and biochars (RS400 and RS700) on root cell death (a) and Al distribution (b) of wheat seedlings. The numbers in the sample names represent the pyrolysis temperatures, and the end numbers represent the amendment sample percentage.

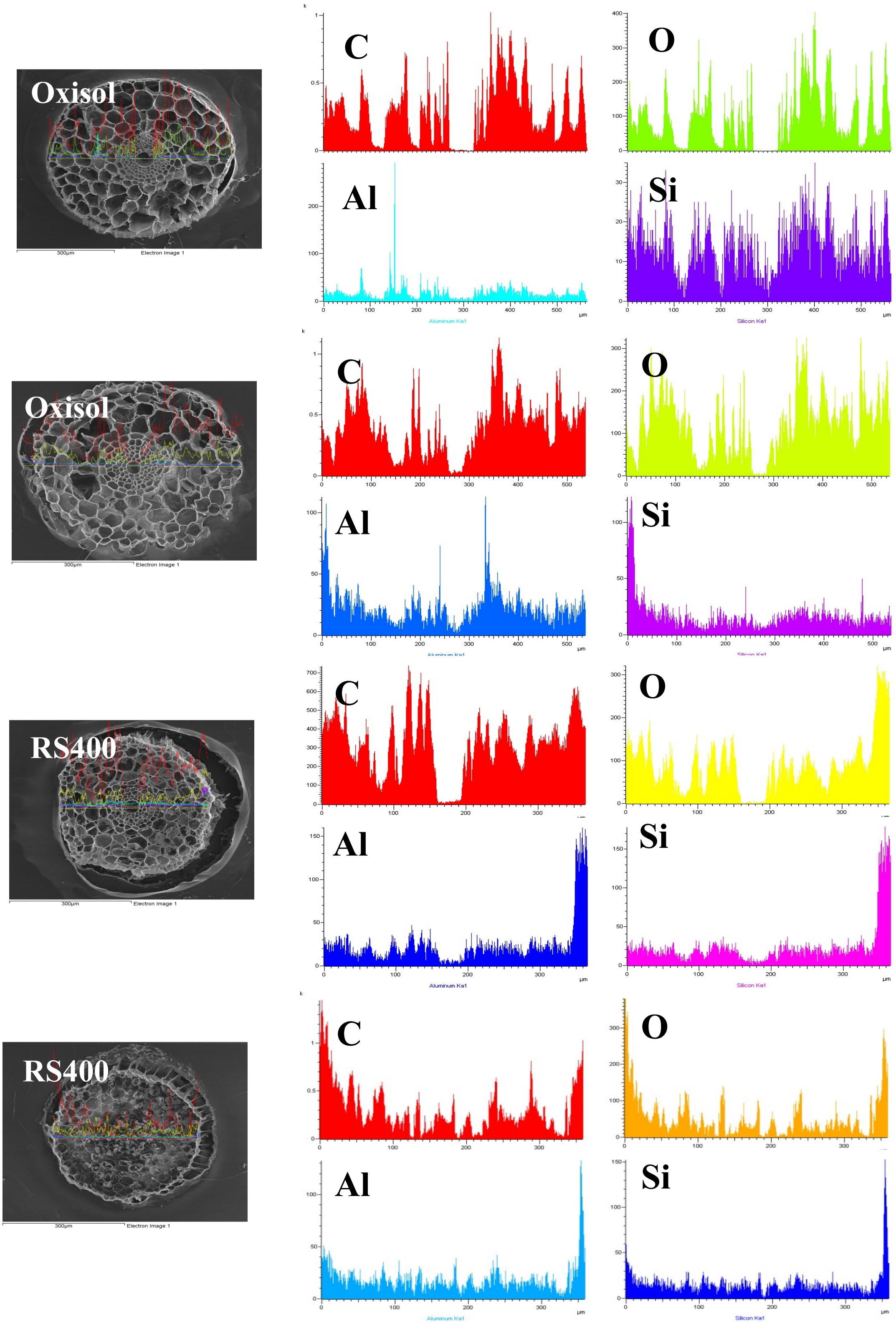

Figure S-2. Effect of acidic soil slurry (oxisol), biochars (RS400) on the elemental mapping of the root tip. The elemental mapping was derived from the line scan of SEM-EDS.
